# Development and internal validation of a new life expectancy estimator for multimorbid older adults

**DOI:** 10.1093/eurpub/ckac130.051

**Published:** 2022-10-25

**Authors:** V Gastens, A Chiolero, D Anker, M Feller, D Bauer, N Rodondi, C Del Giovane

**Affiliations:** 1 Institute of Primary Health Care, University of Bern, Bern, Switzerland; 2 Population Health Laboratory, University of Fribourg, Fribourg, Switzerland; 3 School of Population and Global Health, McGill University, Montreal, Canada; 4 Department of General Internal Medicine, Bern University Hospital, Bern, Switzerland; 5 Departments of Medicine and Epidemiology, University of California, San Francisco, USA

## Abstract

**Background:**

Multimorbidity is highly prevalent among older adults and associated with a shorter life expectancy. Many guidelines recommend tailoring preventive care of multimorbid people according to life expectancy. Indeed, patients with a relatively short life expectancy might not have the time to benefit from a preventive care intervention. Our objective was therefore to develop and internally validate a life expectancy estimator for older multimorbid adults.

**Methods:**

We analysed data of the OPERAM (OPtimising thERapy to prevent Avoidable hospital admissions in Multimorbid older people) cohort study in Bern, Switzerland. 822 hospitalized participants aged 70 years old or more, with multimorbidity (3 or more chronic medical conditions), and polypharmacy (use of 5 drugs or more for >30 days) were included. Our main outcome was time to all-cause mortality assessed during 3 years of follow-up. Candidate predictors included demographic variables (age, sex), clinical characteristics (Charlson-Comorbidity-Index, number of drugs, body mass index, weight loss), smoking, functional status variables (Barthel-Index, falls, nursing home residence), and hospitalization. We internally validated and optimism corrected the model using bootstrapping techniques. We transformed the 3-year mortality prognostic index into a life expectancy estimator using the Gompertz survival function.

**Results:**

At baseline, the participants (58% men) had a median age of 79 years (min: 70; max: 99). They took daily a median of 10 chronic medications (min: 5; max 38). During 3 years of follow-up, 292 participants (36%) died. The analysis is ongoing and results will be presented at the congress.

**Conclusions:**

A life expectancy estimator eventually helps personalising care to prevent under- and overuse of preventive care in the growing older population.

**Key messages:**

- We provide the first life expectancy estimator for older multimorbid adults.
- A life expectancy estimator eventually helps personalising care to prevent under- and overuse of preventive care in the growing older population.

